# Eradication of *Staphylococcus aureus* and *Pseudomonas aeruginosa* Biofilms in Wound Infection Models Using A Novel ML:8‐Loaded Carbopol Gel

**DOI:** 10.1155/ijm/9238905

**Published:** 2026-09-23

**Authors:** Hawraa Shahrour, Daniela Alves Ferreira, Deirdre Fitzgerald-Hughes, James P. O′Gara, Eoghan O′Neill

**Affiliations:** ^1^ Department of Clinical Microbiology, Royal College of Surgeons in Ireland, Dublin, Ireland, rcsi.ie; ^2^ Microbiology, School of Biological and Chemical Sciences, University of Galway, Galway, Ireland, universityofgalway.ie; ^3^ Department of Microbiology, Connolly Hospital, Dublin, Ireland

**Keywords:** antimicrobial resistance, biofilm, fatty acid derivatives, hydrogels, ML:8, wound infection

## Abstract

Chronic wound infections present a significant healthcare challenge, which is compounded by the involvement of resistant biofilms, leading to increased patient morbidity and mortality. Traditional treatments often fail to eradicate biofilms of common chronic wound pathogens such as *Staphylococcus aureus* and *Pseudomonas aeruginosa*. This study is aimed at evaluating the antibacterial and antibiofilm efficacy of ML:8, a fatty acid derivative, in clinically relevant models of chronic wound infections. The antibacterial and antibiofilm efficacy of ML:8 was evaluated against monospecies and polymicrobial biofilms of varying maturity (1, 3, and 5 days old) in in vivo‐like, in vitro wound models. ML:8 demonstrated effective eradication of *S. aureus* and *P. aeruginosa* biofilms, achieving significant (up to 4‐log) reductions in bacterial load. A Carbopol gel formulation containing 10% ML:8 was used, enabling the release of ML:8. The antimicrobial efficacy of the gel was evaluated in an ex vivo porcine wound model, resulting in a significant reduction in biofilms. Furthermore, continuous subculturing experiments showed no development of resistance to ML:8 in vitro. These findings support the use of ML:8 in a gel‐based delivery system as a promising approach for safe and effective management of chronic wound infections involving biofilms and antimicrobial resistance pathogens.

## 1. Introduction

Chronic wound infections pose a significant challenge to healthcare due to their persistent nature and increasing resistance to conventional treatments [1]. These infections are associated with increased patient morbidity and mortality, as well as substantial social and economic costs [1]. A major challenge in treating these infections is the emergence of antimicrobial resistance (AMR), with pathogens like methicillin‐resistant *Staphylococcus aureus* (*S. aureus*) and multiresistant *Pseudomonas aeruginosa* (*P. aeruginosa*) posing significant threats [2]. Furthermore, a primary contributor to the recalcitrant nature of many AMR‐related infections is the presence of biofilms, complex aggregates of bacteria that adhere to surfaces and are enveloped in a protective matrix [3, 4]. Biofilms can consist of multiple bacterial species, with *S. aureus* and *P. aeruginosa* frequently identified as coinfecting and colonizing agents in chronic wounds [2]. This dual‐species interaction further complicates treatment, as the bacteria act synergistically to enhance biofilm resilience and AMR [5]. Furthermore, interactions between *S. aureus* and *P. aeruginosa* have been shown to alter virulence, increase tolerance to antimicrobial therapy, and promote persistence within chronic wounds, making polymicrobial infections particularly challenging to eradicate [6, 7]. In addition to the poor penetration of antimicrobials into the biofilm matrix, biofilms often harbor antibiotic‐tolerant persister cells, which facilitate chronic and relapsing infections [3]. In clinical practice, few antibiotics demonstrate effectiveness against biofilm‐associated infections. Recent advancements in therapeutic agents, for example nanotechnologies, chemical and physical debridement, and antimicrobial dressings offer promising avenues for more effective treatments [8].

Our team has been investigating several bioactive molecules including an antimicrobial peptide and a bacteriophage, which demonstrated strong potential against biofilm‐associated chronic wounds [9, 10]. Among the novel therapies studied for their antibiofilm properties is the fatty acid derivative ML:8, which includes components previously approved for parenteral nutrition and caprylic acid, a fatty acid recognized for its antimicrobial properties, as the primary active ingredient [11, 12]. Fatty acids, such as caprylic acid, have demonstrated efficacy against periodontal pathogens presenting promising results in disrupting biofilms in various clinical contexts [13–15]. Extensive studies performed by our group have previously demonstrated the potential of ML:8 as a therapeutic agent for intravascular catheter‐related infections involving *S. aureus* biofilms [11].

ML:8, although exhibiting potent antimicrobial properties, has been shown to have toxic effects on human cells at high concentrations, limiting its direct application in wound treatment [11]. However, incorporating ML:8 into a Carbopol gel matrix may mitigate these adverse effects and enhance its therapeutic potential for wound care. Carbopol gels are widely used in pharmaceutical formulations due to their excellent properties as bioadhesive, biocompatible, and controlled‐release vehicles [16, 17]. By embedding ML:8 within a gel matrix, localized delivery of the antimicrobial agent may be achieved while potentially reducing direct exposure of surrounding tissues. Localized delivery mediated by the gel facilitates adherence to the wound surface, targets the infection site precisely and reduces systemic exposure and associated side effects [18, 19]. Additionally, the ability of Carbopol gel to maintain a moist wound environment supports healing, while enhancing stability and reducing cytotoxicity, making it a practical and effective option for managing wound infections [20, 21].

Although ML:8 has previously been studied in catheter‐related infections, its application in wound infection models, particularly using ex vivo porcine skin and polymicrobial biofilms, represents a novel and therapeutically relevant advancement.

The aim of this study was to evaluate the antibacterial and antibiofilm activity of ML:8 against monospecies and polymicrobial biofilms of *S. aureus* and *P. aeruginosa* using clinically relevant in vitro, dynamic‐flow, and ex vivo wound infection models, and to assess the feasibility of a Carbopol‐based gel formulation for topical delivery of ML:8.

## 2. Materials and Methods

### 2.1. Antimicrobial Compounds

ML:8 is a proprietary antimicrobial emulsion supplied by Westgate Biomedical, Donegal, Ireland, as disclosed in patent WO2011/061237. It consists of free caprylic acid, also known as n‐octanoic acid, dispersed in Miglyol 812N (neutral triglyceride) in an 80:20 oil phase ratio. When dispersed in a gel carrier at 10% w/w, the gel contains 10 mg/mL of oil phase, with pharmacological properties comparable with an ointment. The gel carrier is 2% w/w Carbopol 974p (Lubrizol Corporation, Wickliffe, Ohio, United States); rheological properties are pH‐dependant and shear‐sensitive: In this composition, it has a viscosity of approximately 6000 cP. The gel is bioadhesive but not biodegradable.

### 2.2. Bacterial Strains and Growth Conditions

The study utilized various bacterial strains, including methicillin‐resistant *S. aureus* (MRSA) isolates USA300 LAC and BH1CC [22], methicillin‐sensitive *S. aureus* (MSSA) strains SH1000 [23] and BH48 [22], as well as *P. aeruginosa* PAO1 (ATCC 15692). The bacteria were cultured aerobically at 37°C, with shaking at 200 rpm in Brain Heart Infusion (BHI, Oxoid, Ireland). Selective media, including mannitol salt agar (Merck, Ireland) for *S. aureus* and MacConkey agar (Merck, Ireland) for *P. aeruginosa*, were used for the reisolation of bacterial strains from mixed‐species biofilms. To simulate in vivo conditions for static antibiofilm testing, Bolton Broth (Fannin, Ireland) was supplemented with 50% bovine heparin plasma (TebuBio, United Kingdom) and 5% laked horse blood (Merck, Ireland). For dynamic biofilm growth, simulated wound fluid (SWF) was prepared as described previously with slight modifications, using 3% v/v fetal bovine serum (FBS) in maximum recovery diluent (Merck, Ireland) [24].

### 2.3. Antimicrobial Susceptibility Testing

Minimal inhibitory concentrations (MICs) were studied using the standard broth microdilution method according to the guidelines by Clinical and Laboratory Standards Institute (CLSI) [25]. Briefly, stock solutions of ML:8 were serially diluted two‐fold in cation adjusted Mueller–Hinton broth (CAMHB) (Merck, Ireland) in a 96‐well round‐bottom polystyrene microplate (ThermoFisher, Ireland). Bacterial suspensions of approximately 10^8^ CFU/mL were prepared directly from plate cultures using a 0.5 McFarland standard (bioMèriux, United Kingdom) in sterile PBS (Merck, Ireland). Afterwards, bacterial suspensions were inoculated in CAMHB with antimicrobial compounds to a final concentration of 10^5^ CFU/mL. Microplates were incubated statically for 24 h at 37°C. Inoculated medium without antimicrobials served as growth control, and medium alone served as sterile control. The lowest concentration of a compound or antibiotic showing no visible growth was recorded as the MIC.

### 2.4. Biofilm Analysis Under Static Conditions

*S. aureus* and *P. aeruginosa* biofilms were formed under static conditions using flat 96‐well polystyrene plates (ThermoFisher, Ireland), as described previously [26]. Briefly, bacterial suspensions were prepared from overnight cultures in Bolton Broth media supplemented with 50% bovine heparin plasma and 5% laked horse blood. A volume of 100 *μ*L of each bacterial suspension was added to each well of a microtiter plate prior to static incubation at 37°C for 1, 3, and 5 days. The media in each well was replaced daily for mature biofilms (at 3 and 5 days). After the initial incubation and washing steps, treatment with ML:8 at varying concentrations of 0.25, 0.5, and 1% (v/v) was introduced into each test well, and the plate was incubated at 37°C for 24 h. Posttreatment, biofilms were washed twice with PBS, and analysis of results was performed. For the polymicrobial biofilms, a 1:1 bacterial suspension ratio of *S. aureus* and *P. aeruginosa* at 10^5^ CFU/mL each was used to inoculate the wells. Fresh media without bacteria served as a negative control and remained in the wells during incubations. The experiments were conducted in triplicate, with independent replicates performed three times on different days.

### 2.5. Measurement of Metabolic Activity of Treated Biofilms

Viability of biofilms after treatment was measured using resazurin‐conversion assay (Merck, Ireland). A stock solution of resazurin at 440 *μ*M was diluted 88 *μ*M in sterile water, and 100 *μ*L of this solution was added to each well. Plates were incubated for further 1 h at 37°C in the dark to determine biofilm metabolic viability after antimicrobial treatment. Fluorescent intensity of the wells was determined using a fluorimeter (Perkin Elmer 2030 Multilabeled Reader Victor 3x), at 544 nm excitation and 590 nm emission. Biofilm metabolic activity was evaluated based on the fluorescence intensity, which is proportional to the number of living cells. Experiments were conducted in triplicate, and independent replicates were performed three times on different days.

### 2.6. Assessment of Biofilm Viable Cells by Enumeration of Colony Forming Units (CFUs)

Following treatment, biofilms formed in the wells were washed twice with 200 *μ*L of PBS. Then, 100 *μ*L of TrypLE solution (Gibco, Dublin, Ireland) was added to each well to resuspend the biofilm. The resuspended biofilms were then 10‐fold serially diluted in sterile PBS. Out of these dilutions, 10 *μ*L aliquots were plated in triplicate on MH agar using the drop method. For polymicrobial biofilms, dilutions were plated on selective agars, mannitol salt agar and MacConkey Agar. The plates were incubated at 37°C overnight. After incubation, CFUs were counted, and results were expressed as log_10_ CFU/mL.

### 2.7. Biofilm Formation and Treatment Using a Biofilm Flow Device

#### 2.7.1. Enumeration of CFUs

The Duckworth biofilm flow device was established and operated following the protocol described by Duckworth et al. [27]. Twelve 10‐mm agar disks (1.5% w/v) were prepared using a French press punch and placed into designated wells in the flow device. Each agar disk was overlaid with a cellulose filter (13 mm, 0.22 *μ*m, Merck Millipore, Ireland) and inoculated with 10 *μ*L of bacterial suspension. The device was incubated at 37°C with a continuous flow of SWF at a rate of 0.322 mL/min for either 24 h or 5 days, using a peristaltic pump as shown in the supporting material file (Figure S1). Following this incubation period, a topical treatment with 1% (v/v) ML:8 was applied for an additional 6 h. The biofilms formed on the cellulose filters were collected and suspended in 1 mL of PBS, and colony counting was performed as detailed for static biofilms.

#### 2.7.2. Confocal Microscopy

The Duckworth biofilm flow device was set up similarly to the colony count method, with slight modifications. Twelve agarose disks were prepared by dissolving 0.3 g of agarose in 20 mL of SWF supplemented with 3% FBS and 0.15% collagen (Merck, Ireland) and mixing with bacterial cultures at 10^8^ CFU/mL. The disks were cut using a 10‐mm French press punch and placed into designated wells in Duckworth device. The experiment was conducted as previously described. After treatment, thin slices of the agarose disks were cut and stained using the bacterial viability LIVE/DEAD BacLight kit (ThermoFisher Scientific, Ireland) following the manufacturer′s instructions. The stained disks were imaged using a confocal laser scanning microscope (Cell Observer Z1, Zeiss, Oberkochen, Germany) equipped with a 63X objective. Images were acquired with the Zeiss software package and processed using ImageJ (ImageJ/Fiji 1.46, National Institutes of Health, United States). Three representative images were obtained per sample group for each experiment.

### 2.8. Investigation of the Potential of ML:8 to Induce AMR In Vitro

This assay was conducted following previously established protocols with some modifications [28]. In short, the in vitro serial passage study involved exposing bacteria, diluted to 10^4^ CFU/mL in CAMHB, to ML:8 at half the MIC. Serial passaging was initiated by harvesting bacterial cells growing at sub‐MIC concentration, inoculating them into fresh CAMHB, and reincubating them with increasing concentrations of ML8 for 18 h at 37°C. This process was repeated daily for 14 days. The assay was performed in independent triplicates.

### 2.9. Ex Vivo Porcine Skin Model to Test Antibiofilm Activity of ML:8 10% Carbopol Gel

#### 2.9.1. Harvesting Porcine Skin Fragments

Porcine skin used in this study was obtained from freshly harvested fragments of healthy pigs that had not undergone any prior treatments or modifications. These samples were donated from other ongoing experiments at the Beaumont Hospital animal facility as part of a recycling initiative aimed at reducing animal use and promoting sustainable research practices.

#### 2.9.2. Wound Infection Model and Treatment

This model was adopted from Andersson et al. [29] with some modifications. Briefly, to form and treat wounds in postmortem porcine skin fragments, the skin was first shaved, rinsed with sterile deionized water, and sectioned into 10 × 6‐cm segments. The surface was disinfected with 70% ethanol and allowed to dry in a laminar flow cabinet. Wounds were created using an 8‐mm sterile biopsy punch to a thickness of 0.5 cm. For infection, wounds were inoculated with 5 *μ*L of bacterial culture (10^4^ CFU) then incubated at 37°C for 24 h. The infected wounds were treated with 10% ML:8‐loaded Carbopol gel (prepared and obtained from Westgate biomedical) for another 24 h as shown in the supporting material file (Figure S2). The 10% ML:8 concentration in the Carbopol gel was selected to ensure sufficient antimicrobial activity in tissue environments, compensating for reduced bioavailability due to gel encapsulation and tissue penetration barriers. Negative controls included uninfected wounds treated with unloaded gel. For analysis, the surface of the wound containing the biofilm was collected, homogenized in sterile PBS and CFU determined by serial dilutions onto MHA plates.

### 2.10. Statistics

Statistical analyses were performed using GraphPad Prism Version 9. Log_10_ transformation of CFU data was applied to ensure normal distribution. For comparisons, two‐way ANOVA with multiple comparisons and Student′s *t*‐test were used as appropriate. Significance thresholds were set at *p* < 0.05, *p* < 0.01, and *p* < 0.001, as indicated in figure legends. All experiments were conducted with three technical replicates per sample and were independently repeated three times (*n* = 3 independent biological experiments). Data are presented as the mean ± standard deviation (SD).

## 3. Results

### 3.1. ML:8 Possess Potent Broad‐Spectrum Activity Against MDR Bacterial Strains

In this study, ML:8 demonstrated potent antibacterial activity against planktonic bacterial strains of *S. aureus* (MRSA and MSSA) and *P. aeruginosa* (PAO1) as shown in Table 1. MIC values were 0.125% (v/v) for all *S. aureus* strains and 0.25% (v/v) for *P. aeruginosa* PAO1, indicating broad‐spectrum activity against both Gram‐positive and Gram‐negative wound‐associated pathogens.

**Table 1:** Minimum inhibitory concentrations of ML:8 against reference and clinical strains of *S. aureus* and *P. aeruginosa.*

Strain	ML:8 (%)
MSSA^a^ SH1000	0.125
MSSA BH48	0.125
MRSA^b^ USA300	0.125
MRSA BH1CC	0.125
*P. aeruginosa* PAO1	0.25

^a^MSSA colon methicillin‐susceptible *Staphylococcus aureus.*

^b^MRSA colon methicillin‐resistant *Staphylococcus aureus.*

### 3.2. ML:8 Eradicates Early and Mature In Vitro Monospecies and Polymicrobial Biofilms

**Figure 1:**
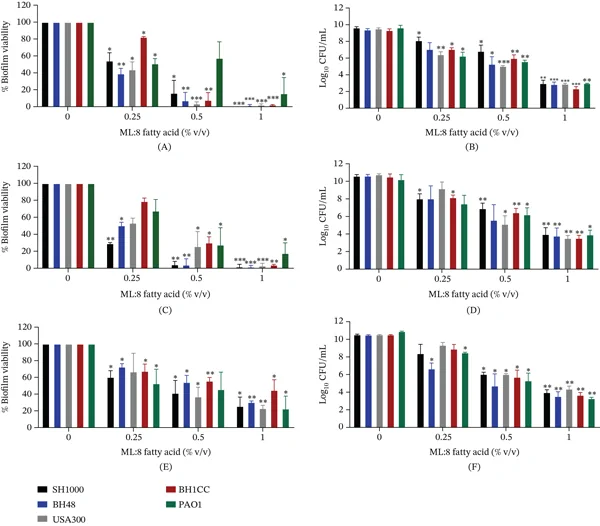
Biofilm eradication by ML:8 (free in solution) against early and mature monospecies biofilms of *S. aureus* and *P. aeruginosa*. Biofilms grown for 1 (A,B), 3 (C,D), and 5 (E,F) days under in vivo‐like in vitro conditions were treated with increasing concentrations of ML:8. Effect of ML:8 on the formed biofilms was determined using resazurin assay (A, C, and E) and colony counts (B, D, and F). Results are the mean ± SD of three independent assays. Obtained results were analyzed using two‐way ANOVA, and statistically significant differences compared with the untreated control are shown, where  ^∗^,  ^∗∗^, and  ^∗∗∗^ indicate a *p* < 0.05, *p* < 0.01, and *p* < 0.001, respectively.

**Figure 2:**
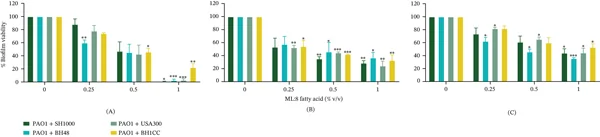
Biofilm eradication by ML:8 (free in solution) against polymicrobial biofilms of *S. aureus* and *P. aeruginosa*. Biofilms grown for 1 (A), 3 (B), and 5 (C) days under in vivo‐like in vitro conditions were treated with increasing concentrations of ML:8. Effect of ML:8 on the formed biofilms was determined using resazurin assay. Results are the mean ± SD of three independent assays. Obtained results were analyzed using two‐way ANOVA, and statistically significant differences compared with the untreated control are shown, where  ^∗^,  ^∗∗^, and  ^∗∗∗^ indicate a *p* < 0.05, *p* < 0.01, and *p* < 0.001, respectively.

**Figure 3:**
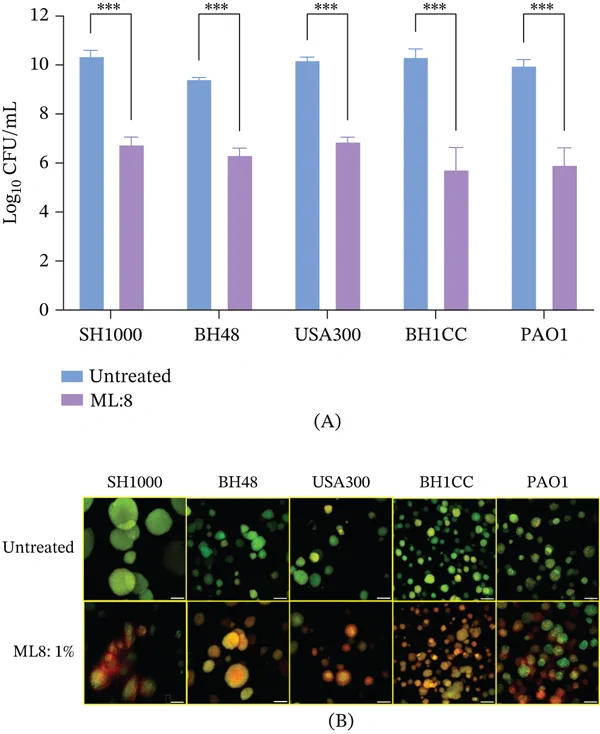
Antibiofilm activity of ML:8 1% free in solution against monospecies biofilms of *S. aureus* and *P. aeruginosa.* Biofilms were allowed to grow for 5 days in Duckworth system before treatment with ML:8 1%. (A) Number of biofilm viable cells presented as the mean of Log_10_ CFU/mL plus standard deviation (±SD) of three independent assays. Obtained results were analyzed using a Student *t*‐test, and statistical differences were significant compared with untreated control,  ^∗∗∗^ indicating a *p* < 0.001. (B) Tridimensional confocal microscopy images of treated and untreated biofilms (Green‐stained cells correspond to viable cells, and red‐stained cells correspond to dead bacterial cells). This experiment was independently repeated three times, and in all the cases, the observations shown here were reproduced. Scale bar corresponds to 100 *μ*m.

**Figure 4:**
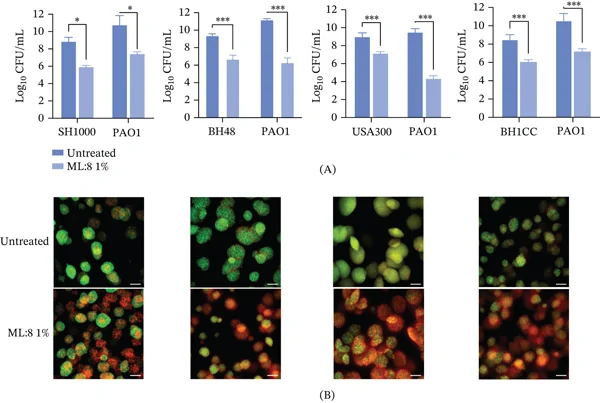
Antibiofilm activity of ML:8 1% free in solution against polymicrobial biofilms of *S. aureus* and *P. aeruginosa*. Biofilms were allowed to grow for 24 h in Duckworth system before treatment with ML:8 1%. (A) Number of biofilm viable cells presented as the mean of Log_10_ CFU/mL plus standard deviation (±SD) of three independent assays. Obtained results were analyzed using a Student *t*‐test, and statistical differences were significant when comparing treated to untreated biofilm,  ^∗^ and  ^∗∗∗^ indicate a *p* < 0.05 and *p* < 0.001, respectively. (A) Tridimensional confocal microscopy images of treated and untreated biofilms (Green‐stained cells correspond to viable cells, and red‐stained cells correspond to dead bacterial cells). This experiment was independently repeated three times, and in all the cases, the observations shown here were reproduced. Scale bar corresponds to 100 *μ*m.

**Figure 5:**
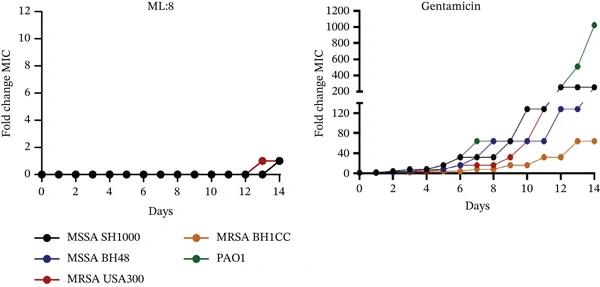
Development of resistance to ML:8 in comparison to gentamicin following serial passage. The data represent the fold change in MIC of ML:8 and gentamicin against *S. aureus* strains (MSSA SH1000, MSSA BH48, MRSA USA300, MRSA BH1CC) and *P. aeruginosa* PAO1 after each passage over a 14‐day duration. Data for ML:8 show zerofold change in MIC for all strains during the first 12 days.

To determine the antibiofilm efficacy of ML:8 under wound‐relevant conditions, monospecies and polymicrobial biofilms of varying maturity were exposed to increasing concentrations of ML:8. Primary biofilm testing was conducted under static conditions in an in vivo‐like environment accomplished by allowing the biofilms to grow in a medium supplemented with plasma and blood. The 24‐h treatments with ML:8 showed a significant concentration‐dependent reduction in monospecies biofilm metabolic activity and viability (Figure 1). The antibiofilm activity of ML:8 was observed across all stages of biofilm maturity, with significant reductions in biofilm viability and bacterial counts in 1‐, 3‐, and 5‐day‐old monospecies biofilms. Reductions of up to 6‐log units were observed following treatment with 1% ML:8. Similar effects were observed in polymicrobial biofilms, where treatment with ML:8 significantly reduced biofilm metabolic activity by up to 80%, demonstrating efficacy against more complex biofilm communities (Figure 2). These data revealed that the optimum dose of ML:8 against biofilms formed by these strains was 1% (v/v). The antibiofilm potential of ML:8 was further investigated against biofilms formed under continuous flow of SWF media in a Duckworth device system. The bacterial isolates were allowed to form monospecies and polymicrobial biofilms for 1 and 5 days in a Duckworth device before being treated with 1% ML:8 for a further 24 h. In the dynamic‐flow wound model, treatment with 1% ML:8 resulted in significant reductions in viable biofilm cells, reaching up to 4‐log reductions compared with untreated controls. This activity was observed in both monospecies and polymicrobial biofilms (Figures 3A and 4A). In independent experiments, biofilms formed were assessed by confocal microscopy using the LIVE/DEAD Baclight viability assay. Consistent with our quantitative data, 24‐h treatment with ML:8 1% resulted in a dominance of dead (i.e., red) cells over viable (i.e., green) cells as visualized in the tridimensional confocal images (Figures 3B and 4B). To evaluate the potential for inducing resistance, continuous subculturing with concentrations of ML:8 one‐fold below the MICs (Figure 5) was conducted over a 14‐day period. Gentamicin was used as a control in these experiments to observe the development of resistance against a conventional antibiotic. Serial passage experiments showed no increase in MIC values of ML:8 for any of the tested strains, indicating no detectable resistance development under the conditions examined. In contrast, resistance to gentamicin emerged after the third passage, highlighting a potential advantage of ML:8 with respect to resistance selection (Figure 5).

### 3.3. Eradication of Ex Vivo Monospecies and Polymicrobial Biofilms by ML:8

**Figure 6:**
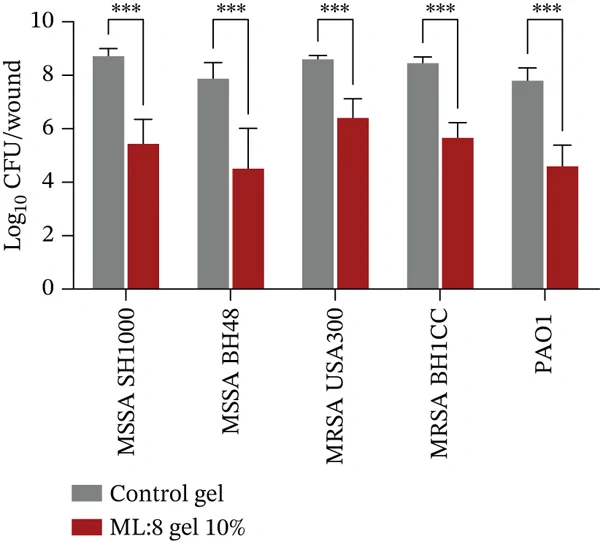
Antibiofilm activity of ML:8 10% Carbopol gel against monospecies biofilms of *S. aureus* and *P. aeruginosa* in a porcine skin infection model. Gel treatment was applied to the wounds after 6 h of incubation with the bacterial strains, and treatment was left for another 24 h at 37°C. The number of viable cells in the wounds was then detected and presented as Log_10_ CFU/wound plus standard deviation (±SD). Analysis was done using a two‐way ANOVA test and multiple comparisons, where the results were shown to be significantly different when comparing treated to untreated biofilms,  ^∗∗∗^ indicating a *p* < 0.001.

**Figure 7:**
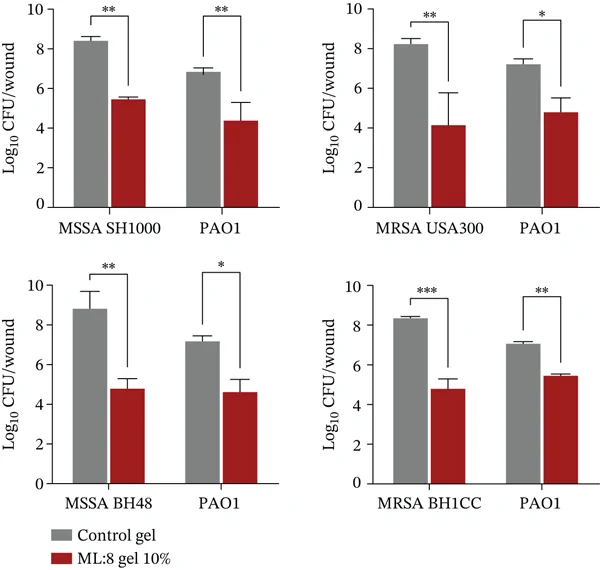
Antibiofilm activity of ML:8 10% Carbopol gel against polymicrobial biofilms of *S. aureus* and *P. aeruginosa* in a porcine skin infection model. Gel treatment was applied to the wounds after 6 h of incubation with the bacterial strains, and treatment was left for another 24 h at 37°C. The number of viable cells in the wounds was then detected and presented as Log_10_ CFU/wound plus standard deviation (±SD). Analysis was done using a two‐way ANOVA test and multiple comparisons, where the results were shown to be significantly different when comparing treated to untreated biofilms,  ^∗^,  ^∗∗^, and  ^∗∗∗^ indicate a *p* < 0.05, *p* < 0.01, and *p* < 0.001, respectively.

Having confirmed potent activity of ML:8 in vitro, the results were translated into an ex vivo porcine wound infection model to simulate incorporation into a wound dressing. We investigated the efficacy of a Carbopol gel incorporating 10% ML:8 in eradicating monospecies and polymicrobial biofilms grown in wounds generated in porcine skin fragments. Bacterial strains were allowed to form biofilms for 24 h before the introduction of treatment. The infected wounds were treated with a 10% ML:8‐loaded Carbopol gel for 24 h. As shown in Figure 6, treatment with 10% ML:8‐loaded Carbopol gel significantly reduced viable bacterial counts in both monospecies and polymicrobial wound biofilms in comparison with unloaded control gel. Reductions of up to 4 log units were observed across all tested strains, demonstrating that antibiofilm activity was retained following incorporation of ML:8 into the gel formulation (Figure 7).

## 4. Discussion

Treating chronic wound infections poses numerous challenges, including the formation of antibiotic‐resistant biofilms, impaired host defenses in individuals with underlying health conditions, persistent inflammation, and the presence of biofilms within the wound [30, 31]. Poor wound‐healing environments characterized by factors such as tissue hypoxia and necrotic tissue further complicate treatment [32]. Therefore, effective management requires a multidisciplinary approach integrating antimicrobial therapy, wound debridement, off‐loading if indicated, and often advanced wound care products [33, 34]. This study has demonstrated effective eradication of mature biofilms where ML:8 possessed potent activity against a spectrum of wound‐associated bacterial strains, including MRSA, MSSA, and *P. aeruginosa.*

The models used in this study mimic the environmental conditions of wound surfaces that support biofilm formation. Several studies have highlighted the importance of simulating actual wound conditions for better clinical relevance [35, 36]. The in vitro biofilm testing was first conducted under static conditions where biofilms were grown under an in vivo‐like environment for 1, 3, and 5 days. ML:8 at 1% (v/v) inactivated early and late/mature biofilms showing a significant reduction in bacterial counts. At this same concentration, ML:8 demonstrated a significant reduction in biofilms grown in the Duckworth device under dynamic flow. This system closely mimics the physiological environment present in chronic wounds by allowing a continuous flow of nutrients to be absorbed at the biofilm site.

Understanding the MICs of novel antimicrobial agents against planktonic and biofilm growth is critical for assessing their potential for progression to clinical development. Biofilms are shown to be up to 1000 times more resistant than planktonic cells and require at least 50 times the MIC of antimicrobials along with sometimes prolonged exposure times [37–39]. Similarly, Stuermer et al. [40] reported that octenidine hydrochloride and sodium hypochlorite were able to eradicate planktonic cells of *S. aureus* and *P. aeruginosa* in seconds but required 48–72 h of exposure to affect biofilms formed in a wound‐like environment. In our study, ML:8 inhibited bacterial growth at 0.125% (v/v) and 0.25% (v/v) and eradicated biofilms at 1% (v/v). Previous reports also indicated that mixed species biofilms in the wound bed exhibit increased AMR [41]. Therefore, mimicking these conditions in our experiments provides more reliable predictions of clinical performance.

Images taken for the biofilms treated with ML:8 have shown its efficacy in inducing biofilm cell death, with a predominance of dead cells (red) over viable cells (green). ML:8′s antimicrobial activity is likely multimodal. The active ingredient, caprylic acid, has been shown to disrupt bacterial membranes, interfere with metabolic pathways, and inhibit quorum sensing [15]. Its efficacy against periodontal pathogens and biofilms supports its broad‐spectrum potential [42, 43]. Additionally, analysis of the bacterial transcriptional response to ML:8 revealed a decrease in expression of genes associated with adherence and exoenzymes, whereas toxin gene expression was both upregulated and downregulated (unpublished data; S. Hogan thesis). Although the available evidence supports a multimodal mode of action, further experimental studies are required to fully characterize and confirm the precise mechanism(s) by which ML:8 exerts its antimicrobial and antibiofilm effects. The possibility of having multiple modes of action supports the reduced likelihood of resistance emergence. This was supported by the serial subculturing experiments performed in this study, which showed no detectable resistance development in *S. aureus* or *P. aeruginosa* under the conditions tested. This is a critical advantage of ML:8 over conventional antibiotics, which are often associated with rapid resistance development.

Our prior studies have shown that ML:8 does not induce cytokine expression, indicating no immune activation at tested concentrations. Furthermore, ML:8′s cytotoxicity and hemolytic effects were examined previously. Cytotoxicity was observed in THP‐1 and HaCaT cells at concentrations effective against biofilms (1% (v/v)), and hemolysis occurred only at higher concentrations. Importantly, ML:8′s cytotoxicity and hemolysis levels were comparable with those of existing clinical agents, such as ethanol and Duralock‐C [11]. These findings, along with ML:8′s limited immunogenicity, suggest it may be a safe and effective option for topical application in wound infection management.

To facilitate topical administration of ML:8, we formulated a stable Carbopol‐based gel. Carbopol was selected as a gel carrier due to its bioadhesive properties, formulation stability, and widespread use in topical drug delivery systems [44, 45]. Previous studies have demonstrated its suitability for delivering antimicrobial agents in wound‐related applications [46, 47]. Compared with other hydrogels such as chitosan, alginate, and hydroxyethyl cellulose, Carbopol offers superior bioadhesion, formulation ease, and stability [48, 49]. Additionally, caprylic acid, the active component of ML:8, is generally recognized as safe (GRAS), and its controlled release via Carbopol mitigates irritation risks, as supported by FDA safety assessments [50, 51]. In the present study, incorporation of ML:8 into a Carbopol matrix enabled topical administration while maintaining substantial antibiofilm activity in the ex vivo model.

We translated our in vitro findings into a clinically relevant setting by evaluating a 10% ML:8 Carbopol gel in an ex vivo porcine wound infection model. Although 1% ML:8 was effective in vitro, a higher concentration (10%) was used in the gel to ensure therapeutic efficacy in the ex vivo wound model. Porcine skin was selected because its anatomy and physiology closely resemble those of human skin, making it an excellent proxy for human dermatological studies. This ensures that the results obtained are predictive of human responses thereby enhancing the reliability of the treatment′s efficacy and safety profile [52]. Using ex vivo skin fragments offers the advantage of naturally occurring wound bed components and allows for precise control over the dose and duration of treatment application [53]. Consistent with previous findings showing stable *P. aeruginosa* and *S. aureus* infections in porcine skin [29], we established monospecies and polymicrobial biofilms using clinically relevant strains. Treatment with the ML:8 gel achieved up to 4‐log reduction in viable bacteria within wound bed, demonstrating strong potential for managing chronic wound infections. This study expands the application of ML:8 beyond catheter models, demonstrating potent antibiofilm activity in an ex vivo wound infection model. Although these findings support the potential of ML:8 as a topical antibiofilm treatment, the model used in this study does not permit evaluation of wound‐healing outcomes, as it lacks key physiological features of living tissue, including blood perfusion, immune responses, and tissue remodeling, and it also lacks active antibiotic or topical antimicrobial comparators. Future translational validation should include comparison with active antibiotics and established topical antimicrobials, ideally in in vivo wound models that permit simultaneous evaluation of antimicrobial efficacy, local irritation, tissue compatibility, wound healing, and safety.

## 5. Conclusions

This study demonstrates the strong antimicrobial potential of ML:8 against clinically relevant wound pathogens in both in vitro biofilm systems and ex vivo porcine wound infection model. ML:8 exhibited potent activity against planktonic MRSA, MSSA, and *P. aeruginosa*, and effectively eradicated mature monospecies and polymicrobial biofilms. The concentration required for biofilm inactivation (1% v/v) aligns with the elevated antimicrobial thresholds typically observed for biofilm‐associated infection, highlighting ML:8′s clinical potential. Continuous subculturing experiments revealed no evidence of resistance development in *S. aureus* or *P. aeruginosa*, suggesting a favorable resistance profile compared with conventional antibiotics. The ex vivo porcine skin model further confirmed the antibiofilm activity of ML:8 in a clinically relevant wound infection setting. However, the present study was not designed to evaluate wound‐healing efficacy, tissue regeneration, or safety of the final gel formulation. Future in vivo studies will be required to assess these outcomes and to further establish the translational potential of ML:8‐loaded Carbopol gel.

## Author Contributions

H.S.: visualization, investigation, formal analysis, conceptualization, methodology, data curation, validation, writing—original draft preparation, writing—review and editing. D.A.F.: investigation, formal analysis, methodology, validation, writing—review and editing. D.F‐H.: conceptualization, methodology, writing—review and editing. J.P.O.: conceptualization, methodology, writing—review and editing. E.O.: methodology, funding acquisition, supervision, writing—reviewing and editing.

## Funding

This study was supported by Health Research Board (10.13039/100010414; 19374A01).

## Ethics Statement

The studies involving animal fragments were conducted in accordance with the local legislation and institutional requirements.

## Conflicts of Interest

The authors declare no conflicts of interest.

## Supporting information


**Supporting Information** Additional supporting information can be found online in the Supporting Information section. Figure S1: The figure represents a labeled illustration of the biofilm formation in the Duckworth device flow system [10]. Figure S2: The figure shows images of the infected wounds formed in the porcine skin model treated and untreated with the gel.

## Data Availability

All data generated or analyzed during this study are included in this published article (and its Supporting Information files); further inquiries can be directed to the corresponding author.
