# Supplementary material for: Eradication of Staphylococcus aureus and Pseudomonas aeruginosa Biofilms in Wound Infection Models Using A Novel ML:8‐Loaded Carbopol Gel
Source: Int J Microbiol. 2026 Sep 23;2026:9238905. doi: 10.1155/ijm/9238905 (PMC13601853; doi:10.1155/ijm/9238905)
Supplement: Supplementary file 1 [file IJM-2026-9238905-s001.docx]

**Figure S1*.* Biofilm formation in duckworth device flow system.** A) Image showing the 3D printed Duckworth deivice and the connected system where the fresh media flow into the device and waste flows out. B)Ilustration of the duckworth system connections [10].

**Figure S2*. Ex vivo* porcine skin wound infection model.** The left image shows the wound formed in the skin using punch biopsy. Right image shows gel treatment application to the wounds after being infected and incubated for 6h.
